# Route Optimization for UVC Disinfection Robot Using Bio-Inspired Metaheuristic Techniques

**DOI:** 10.3390/biomimetics9120744

**Published:** 2024-12-05

**Authors:** Mario Peñacoba, Eduardo Bayona, Jesús Enrique Sierra-García, Matilde Santos

**Affiliations:** 1 Department of Digitalization, University of Burgos, 09001 Burgos, Spain; ebayona@ubu.es; 2 Institute of Knowledge Technology, University Complutense of Madrid, 28040 Madrid, Spain; msantos@ucm.es

**Keywords:** bio-inspired algorithms, ultraviolet radiation (UVC), disinfection, mobile robots, Gazelle optimization algorithm (GOA), whale optimization algorithm (WOA), bat optimization algorithm (BA), particle swarm optimization (PSO)

## Abstract

The COVID-19 pandemic highlighted the urgent need for effective surface disinfection solutions, which has led to the use of mobile robots equipped with ultraviolet (UVC) lamps as a promising technology. This study aims to optimize the navigation of differential mobile robots equipped with UVC lamps to ensure maximum efficiency in disinfecting complex environments. Bio-inspired metaheuristic algorithms such as the gazelle optimization algorithm, whale optimization algorithm, bat optimization algorithm, and particle swarm optimization are applied. These algorithms mimic behaviors of biological beings such as the evasive maneuvers of gazelles, the spiral hunting patterns of whales, the echolocation of bats, and the collective behavior of flocks of birds or schools of fish to optimize the robot’s trajectory. The optimization process adjusts the robot’s coordinates and the time it takes to stops at key points to ensure complete disinfection coverage and minimize the risk of excessive UVC exposure. Experimental results show that the proposed algorithms effectively adapt the robot’s trajectory to various environments, avoiding obstacles and providing sufficient UVC radiation exposure to deactivate target microorganisms. This approach demonstrates the flexibility and robustness of these solutions, with potential applications extending beyond COVID-19 to other pathogens such as influenza or bacterial contaminants, by tuning the algorithm parameters. The results highlight the potential of bio-inspired metaheuristic algorithms to improve automatic disinfection and achieve safer and healthier environments.

## 1. Introduction

In recent years, the need to ensure healthy and safe environments has gained unprecedented importance, particularly in response to the COVID-19 pandemic. Automated disinfection has emerged as a key technological solution to mitigate the spread of microorganisms in various spaces. In this context, mobile robots equipped with ultraviolet (UVC) disinfection lamps have emerged as a promising option due to their ability to navigate complex environments and effectively deactivate pathogens such as SARS-CoV-2, the virus responsible for COVID-19, as well as other viruses and bacteria. However, the effectiveness of these robots largely depends on their ability to navigate the environment optimally, maximizing disinfection coverage and minimizing the risk of collisions with obstacles.

This paper addresses that problem using an approach based on bio-inspired metaheuristic algorithms to optimize the trajectories of a differential mobile robot equipped with a UVC lamp, with the goal of maximizing the efficiency of automatic disinfection in different spaces. The algorithms evaluated in this study are the gazelle optimization algorithm (GOA), the whale optimization algorithm (WOA), the bat optimization algorithm (BA), and the particle swarm optimization (PSO). These algorithms have been selected for their ability to address complex nonlinear optimization problems. Each of them mimics mechanisms of biological beings to find solutions that balance multiple factors. In particular, the GOA is inspired by the evasive behavior of gazelles when fleeing from predators seeking survival. The ability of gazelles to avoid obstacles and adjust their speed and direction is especially useful for optimizing trajectories in environments with obstacles [1]. The WOA is based on the hunting strategy of humpback whales, simulating the spiraling motion they use to surround and catch their prey [2]. This technique allows the WOA to balance global exploration of the solution space with local exploitation, making it useful for finding optimal routes in scenarios with multiple constraints. The BA is inspired by the echolocation of bats, which adjust their search behavior based on the perceived distance to objects. This algorithm is particularly useful for dynamically adjusting the robot’s trajectory, allowing it to “sense” and avoid obstacles in real time, making it an ideal solution for complex environments [3]. Finally, the PSO is inspired by the collective behavior of flocks of birds and schools of fish, optimizing individual solutions (particles) through cooperation and information sharing. Particles adjust their trajectories based on both their own experiences and those of the group, facilitating rapid convergence towards optimal route solutions, especially in large and weakly constrained environments [4].

These algorithms were used to optimize the robot’s disinfection routes in different spaces, determining the coordinates of the passage points and the stopping time at each of them. These parameters must guarantee an obstacle-free route and a sufficient, but not excessive, exposure time to radiation to achieve total deactivation of microorganisms, while protecting the environment from overexposure.

The main challenge of this study is that the environments to be disinfected is not homogeneous and may present different levels of difficulty due to the presence of obstacles or hard-to-reach areas. In this context, bio-inspired algorithms have proven to be highly effective in dynamically adjusting routes based on the specific characteristics of the space, avoiding collisions and ensuring full exposure of all surfaces to UVC radiation. It is important to note that route optimization not only affects the robot’s trajectory but also crucial aspects such as the time spent at each point, since the robot needs to stop at certain locations for enough time to ensure the elimination of microorganisms, in this case the SARS-CoV-2 virus [5].

The results obtained demonstrate that, after optimization, the robot is able to navigate through different spaces efficiently, avoiding collisions with obstacles and ensuring complete disinfection. This work experiments with radiation levels suitable for disinfecting COVID-19, but this solution can easily be extended to other viruses, such as influenza, or any pathogen susceptible to UVC radiation. In addition, the proposed methodology allows its application in environments of varying complexity, dynamically adjusting the robot’s routes to the specific conditions of the space in question.

The main contributions of this work can be summarized as follows:

- The development of an innovative methodology for complete coverage path planning (CCPP) optimization based on polyline optimization. The path planning of a mobile robot equipped with UVC disinfection technology is optimized, considering obstacles in the environment;
- The design of a sequential fitness function that reduces collisions, ensures complete coverage, adjusts radiation dose, and minimizes time. This fitness function has been compared to previous work, giving superior results in the context of pathogen disinfection;
- The systematic simulation of experiments to validate the performance of the solutions. The optimized trajectories significantly reduce travel time and improve coverage, ensuring that the entire target area meets the required disinfection threshold;
- The comparison of four bio-inspired optimization techniques: the GOA, WOA, BA, and PSO. The results indicate that while they all work well to solve this CCPP problem, the GOA offers the best performance in simpler environments. However, the PSO shows very good results in more complex scenarios, effectively refining the path to achieve optimal disinfection coverage.

The organization of the rest of this paper is as follows: Section 2 summarizes the state of the current technology. Section 3 describes the use case, including the experimental setup for the disinfection application. Section 4 presents and compares the results obtained with the four proposed bio-inspired optimization techniques, evaluating the strengths and weaknesses of each method in terms of disinfection capabilities, time, distance, and overall efficiency. This paper concludes with the main findings and suggestions for future work.

## 2. Related Works

Mobile robot trajectory optimization has been and remains a very active research field in recent decades, driven by the need to develop more efficient and safer autonomous systems for various applications such as surveillance, logistics, and disinfection. Traditionally, trajectory planning methods have been based on deterministic approaches, with algorithms such as A*, Dijkstra, and rapidly exploring random tree (RRT) [6]. These studies collectively review the evolution of trajectory planning methods, highlighting classical deterministic approaches (e.g., A* and Dijkstra), heuristic strategies, and their applications in various domains, such as mobile robots [7], underwater vehicles [8], and subsurface systems [9]. Furthermore, they emphasize the strengths and limitations of these algorithms in different environments, establishing a benchmark for evaluating emerging techniques, such as bio-inspired metaheuristic algorithms. These algorithms, which are effective in structured and simple environments, present limitations in more complex or dynamic environments, with uncertainty and where obstacles can change unpredictably, as presented in [10].

For example, the A* algorithm has been successfully used in path planning for mobile robots in static environments. In a study by Wang et al. [11], A* was applied to robot navigation in structured areas, showing robust performance in path optimization and avoiding fixed obstacles. However, the lack of adaptability of A* to dynamic environments limits its application in more realistic and changing scenarios.

Similarly, Dijkstra’s algorithm has been widely used in networking and logistics applications, where finding the shortest path in a graph is required. For example, Vivaldini et al. applied Dijkstra’s algorithm for path planning of logistics robots in warehouses [12]. Although the approach guaranteed optimal paths in static environments, it showed limitations in scalability and in its ability to adapt to real-time changes. Another example is the work of Luo et al. [13], which presents an extension of Dijkstra’s algorithm for optimal path planning on complex surfaces. The proposed method improves the efficiency of path finding, allowing mobile robots to navigate more effectively on uneven surfaces and in complex environments.

The RRT algorithm, in turn, has proven to be an effective technique for path planning in environments where speed and exploration are essential. Unlike A* and Dijkstra, which focus on finding optimal paths in predefined graphs, RRT stands out for its ability to quickly explore large, unstructured spaces, as shown in [14]. This makes it especially useful in scenarios where obstacles are numerous or constantly changing. For example, in the work of Qi et al. [15], RRT was used for path planning of autonomous vehicles in dynamic environments, effectively avoiding obstacles without the need to continuously recalculate the entire path, as would be necessary with deterministic algorithms.

These algorithms have also been hybridized with more novel techniques, such as in the work of Qiang et al. [16], where emergency evacuation routes were optimized using a hybrid Dijkstra–GA. Similarly, in Kiani et al. [17], the adapted RRT combined RRT with metaheuristic algorithms. This approach is particularly interesting as it leverages RRT’s rapid exploration capability to generate initial solutions, and then employs metaheuristic techniques to optimize the obtained trajectories, improving precision and reducing the length of routes in complex three-dimensional environments. The use of this combination overcame the limitations of traditional RRT, which does not guarantee optimality, while the metaheuristics introduced adaptability and efficiency in the final solution. This work highlights how the integration of metaheuristic techniques, such as the particle swarm optimization (PSO) or genetic algorithm (GA), with traditional algorithms, is revolutionizing trajectory planning, especially in problems involving large search spaces and multiple constraints.

However, in recent years the increasing complexity of the environments in which mobile robots must operate has led to the need for more flexible and adaptive approaches to path planning [18]. As mentioned above, traditional methods such as A*, Dijkstra, and RRT, while effective in controlled and static scenarios, encounter difficulties in dynamic and unstructured environments. To overcome these limitations, metaheuristic algorithms have gained prominence in mobile robot path optimization, as detailed in [19].

Metaheuristic algorithms are a group of optimization techniques that allow for the exploration of large search spaces, providing efficient solutions even for highly complex problems [20]. Unlike deterministic algorithms, metaheuristic strategies do not guarantee finding the optimal solution, but they are able to find sufficiently good solutions in a reasonable amount of time, making them an ideal tool for real-time path planning. Among the most notable ones are simulated annealing [21], tabu search [22], and evolutionary algorithms such as genetic algorithms (GAs) and particle swarm optimization (PSO). Other techniques, such as pattern search (PS), gray wolf optimization (GWO), ant colony optimization (ACO), and artificial bee colony (ABC), have also been successfully applied to autonomous robot navigation problems.

Bio-inspired algorithms are a subset of metaheuristic techniques and have emerged as a highly efficient solution for trajectory optimization [23]. These algorithms mimic natural processes observed in biology and physics, allowing robots to adapt to their environment in a flexible and efficient manner. Their popularity has grown due to their ability to address nonlinear optimization problems in dynamic environments, where conditions are continuously changing [24].

One of the most widely used algorithms is the particle swarm optimization (PSO), which is inspired by the collective behavior of flocks of birds or schools of fish [25]. This algorithm optimizes the position of a set of particles within a search space, emulating the behavior of individuals moving collectively towards a common goal [26]. It has been applied to a wide range of problems, from optimizing chemical reactions in thermal engines [27] to enhancing the efficiency of mobile robots navigating environments with multiple obstacles [28].

Another very commonly used technique is the genetic algorithm (GA), which is based on the principles of biological evolution, such as natural selection, mutation, and gene crossover. GA has been used for robot trajectory optimization for decades, providing robust and adaptive solutions as the environment changes. As an example, in the work of Lamini et al. [29], GA was used to optimize robot trajectories in various simulated environments, achieving efficient and adaptive navigation.

In addition to what could be considered as “traditional bio-inspired metaheuristic techniques”, new techniques have been proposed recently, such as the whale optimization algorithm (WOA), the bat algorithm (BA), and the gazelle optimization algorithm (GOA), each of which emulates different behaviors of living beings allowing for efficient exploration and exploitation of the search space. The WOA, for example, is inspired by the hunting behavior of humpback whales. Chhillar and Choudhary successfully applied it in the optimization of mobile robot trajectories [30], balancing global search with precise local exploitation. The BA, on the other hand, is inspired by the echolocation of bats and has also been proven useful for navigating in complex environments, where obstacle detection and precise navigation are critical [31].

On the other hand, after reviewing some advances in trajectory optimization using bio-inspired algorithms, it is also relevant to refer to the other key aspect of this study: disinfection using ultraviolet radiation (UVC) [32]. UVC-based disinfection has gained considerable importance in recent years, particularly in the context of the COVID-19 pandemic, where the need to maintain healthy and pathogen-free spaces has driven the development of new automatic technologies [33,34].

UVC radiation is very effective in deactivating microorganisms such as viruses, bacteria, and fungi by penetrating their cell membranes and damaging their DNA or RNA, preventing their ability to replicate. This disinfection method has been studied in surface and air applications, with a high level of effectiveness [5]. There are applications of this method in hospital environments, public transport [35], offices [36], dental clinics [37], etc. Recently, it has been used with autonomous robotic systems [38]. In this work, a path optimization method for automated UVC disinfection was studied in simple environments with a low number of obstacles, simulating specific hospital spaces. The results showed the potential of autonomous systems in the fight against infectious diseases.

## 3. Definition of the Use Case

As already mentioned, in the fight against the spread of pathogens, automated disinfection has become increasingly relevant. These types of solutions are especially useful in closed and high-traffic environments such as hospitals, offices, shopping malls, public transport systems, or educational centers, where traditional cleaning methods are less effective in terms of time and coverage or require more manpower and often do not guarantee the complete elimination of microorganisms on hard-to-reach surfaces or large areas. In addition, these methods are more prone to human error, which can compromise the quality of the disinfection process—a critical concern in the context of the spread of pathogens such as SARS-CoV-2.

In this scenario, mobile robots equipped with ultraviolet radiation (UVC) systems emerge as an advanced technological solution that not only ensures the elimination of microorganisms but also allows this to be carried out autonomously, without the need for continuous human intervention, avoiding possible health risks. UVC disinfection systems are highly effective as ultraviolet C radiation can penetrate cell membranes and deactivate the genetic material of viruses, bacteria, and other pathogens, preventing their reproduction and spread. This makes UVC disinfection an ideal method to complement or replace traditional cleaning strategies in areas where the presence of pathogens can pose a significant health risk.

### 3.1. Disinfection System

**Figure 1:**
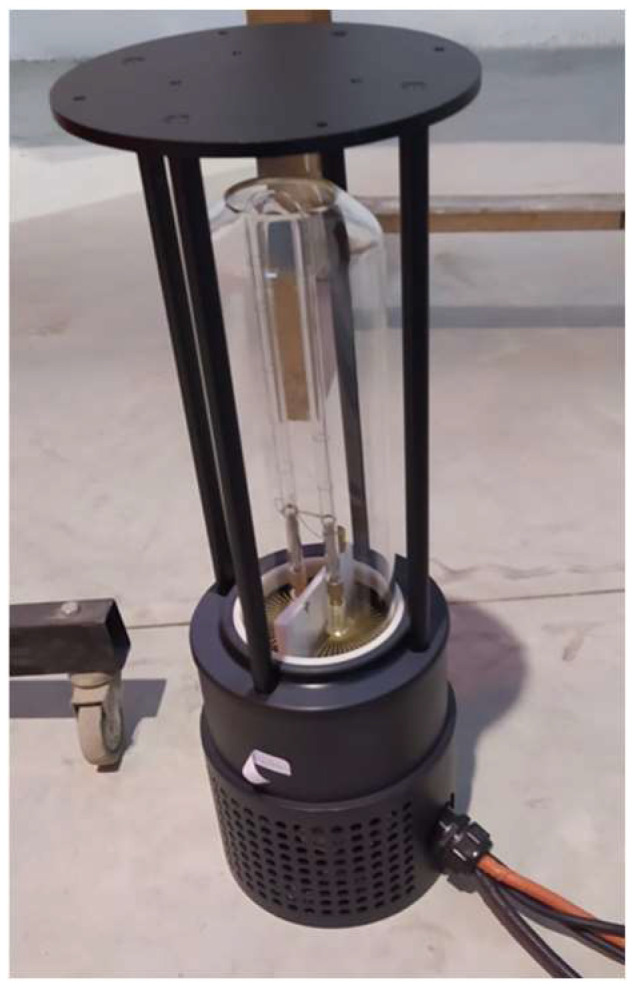
A 207J High-energy pulse UVC lamp.

In this work, a mobile robot is used to optimize disinfection in complex and challenging environments, characterized by the presence of obstacles of various shapes and sizes, as well as in different environments, ranging from large open areas to small spaces or rooms. The robot is equipped with a 207J high-energy pulse UV lamp, a high-tech device specifically designed to emit intense pulses of UVC radiation. This type of UV radiation, particularly in the 200–280 nm range, is highly effective in deactivating microorganisms. The 207J lamp is capable of generating extremely powerful energy pulses, allowing for rapid and effective disinfection in a short exposure time. This is particularly beneficial in applications where it is critical to cover large areas quickly, such as hospitals or spaces with a constant flow of people (Figure 1). Combined with the robot’s autonomous navigation capabilities, this offers a comprehensive solution to disinfect large and complex spaces efficiently, accurately, and safely.

**Figure 2:**
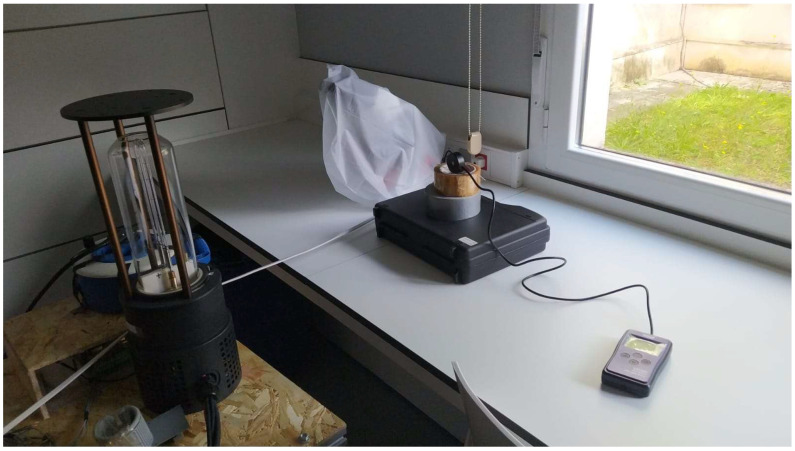
A 207J High-energy pulse UVC lamp with radiation measured at 1 m distance.

The disinfection lamp shown in Figure 1 emits radiation with an intensity of 4.5 J/m² when placed one meter away for one second, with the exposure being perpendicular to the radiation source (as shown in Figure 2).

Furthermore, it has been assumed that the intensity of this radiation decreases proportionally with the square of the distance [39]. Therefore, to achieve a SARS-CoV-2 deactivation rate of 99%, the target radiation level has been set at 500 J/m^2^. Based on these assumptions, the radiation spread of the lamp was calculated using Equation (1).

(1) $$R=\frac{P_{L}\times k\times t}{D^{2}}$$

In Equation (1), $P_{L}$ [W] is the lamp power; *k* $\left[m^{2}/J\right]$ is a scattering constant, which depends on the medium to be disinfected (k=0.71); t [s] is the disinfection time; and D [m] is the distance between points.

### 3.2. Mobile Robot

**Figure 3:**
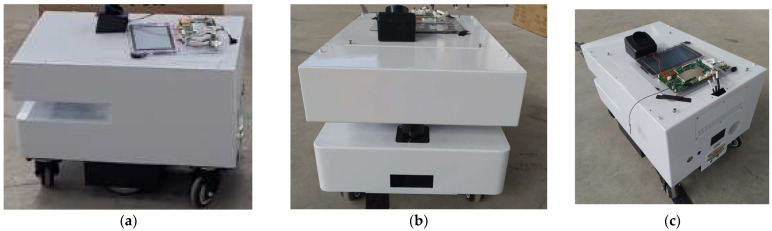
Hussar Robot; side view (**a**), front view (**b**), and isometric view (**c**).

The “Hussar” robot, a compact and versatile robot with dimensions of 450 mm long, 450 mm wide, and 317 mm high, has been used, providing great adaptability to operate in a wide variety of environments, especially those where space is limited, or there are multiple obstacles. Its small size allows it to move easily through narrow corridors, between furniture or equipment, making it ideal for applications in hospitals, offices, educational centers, or even public transport systems [40]. Thanks to this compact design, the robot can perform agile and efficient maneuvers, avoiding potential collisions with both stationary and moving obstacles (Figure 3).

The Hussar robot can move at speeds ranging from 0.2 to 0.8 m/s. However, the version available in the laboratory has a speed limitation of 0.5 m/s. Therefore, in the experiments, the robot speed has been considered constant and has been set to a maximum of 0.5 m/s.

The Hussar robot is also equipped with an Android 5.1 operating system, allowing it to communicate with the on-board computer through various platforms, such as a PC, Raspberry Pi, or Arduino, using the Robot Operating System (ROS Kinetic) [40]. This multi-device integration capability provides great flexibility in controlling the robot and allows for the advanced programming of routes and tasks, optimizing its performance in a wide range of applications.

Another key feature of the robot is its high level of autonomy that allows it to operate continuously for up to 6 h. This is important in disinfection applications where large areas need to be covered or long tasks need to be performed, without the need for constant intervention to recharge the robot. This extended autonomy significantly reduces downtime, maximizing its operational efficiency compared to other systems that require more frequent recharges. It is also equipped with an advanced automatic recharging system, which allows it to autonomously return to its charging station once the battery reaches low levels. This recharging system not only minimizes human intervention but also optimizes the robot’s operational efficiency by ensuring that it is always operational to resume disinfection tasks in the shortest possible time. This ensures continuity in disinfection cycles, which is especially valuable in environments where sanitization is required, such as hospitals or high-traffic areas.

Lastly, this robot has an advanced navigation capability, which allows it to accurately follow pre-set routes. Equipped with intelligent sensors and controllers, the robot can detect obstacles in real time and autonomously adjust its path to avoid collisions, enabling it to respond to unexpected changes in the environment.

### 3.3. System Architecture

**Figure 4:**
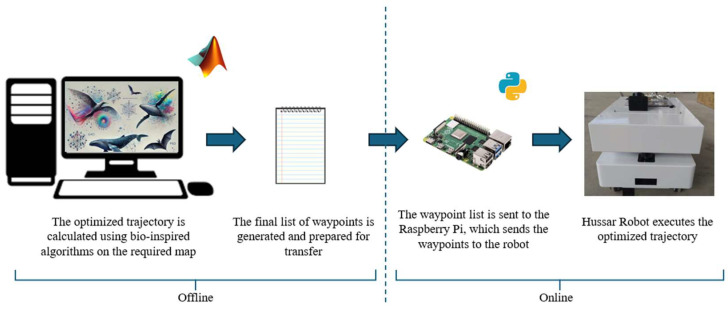
Workflow of the optimized trajectory execution process.

The workflow for achieving an optimized trajectory operates as follows: first, the environment is modeled as a map, and the bio-inspired algorithms are implemented in MATLAB 2021b to compute the optimized trajectory. Once the final waypoints of the optimized trajectory are obtained, they are transferred to a Raspberry Pi. The Raspberry Pi runs a Python script that sequentially sends commands to the real robot, enabling it to follow the calculated trajectory within the actual environment. This process is schematically illustrated in Figure 4.

**Figure 5:**
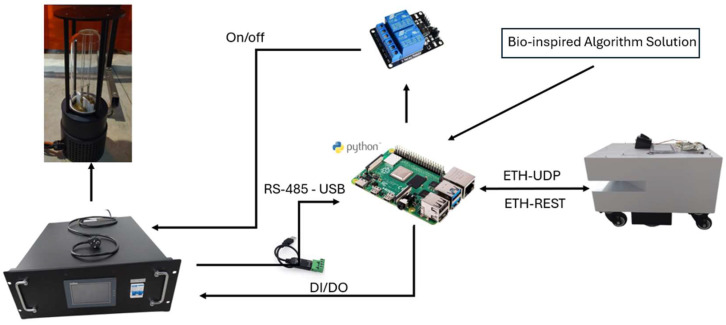
System architecture.

Furthermore, to effectively deactivate the targeted virus or microorganism, the system architecture diagram is presented in Figure 5. This diagram illustrates the control and management of both the robot and the UVC lamp (model 207J), which are coordinated through a Raspberry Pi 4 (Raspberry Pi Foundation, Cambridge, UK).

The Hussar robot, developed by Reeman Robotics (Shenzhen, China), connects to the controller via ETH-UDP and ETH-REST communication links. These Ethernet-based protocols enable the remote transmission of commands and ensure precise delivery of movement and positioning instructions, allowing accurate navigation in specific areas within the environment for targeted disinfection. To achieve this, the robot is equipped with a 3D LIDAR sensor, which generates detailed maps of its surroundings. Using these maps together with real-time data from the LIDAR allows the robot to autonomously navigate in complex environments, detecting and avoiding obstacles.

In addition to its mapping capabilities, the navigation algorithm is integrated into the Raspberry Pi. The robot continuously communicates its current position to the control unit, which in turn calculates and commands the next target position on the navigation path. This feedback loop ensures that the robot follows the intended route.

The UVC lamp is connected to the system via a relay module that allows the lamp to be switched on and off remotely. This relay is linked to the Raspberry Pi, which controls the lamp via a digital interface (DI/DO). Communication between the Raspberry Pi and the lamp control unit is facilitated by a USB to RS-485 converter. This setup allows the UVC lamp to be activated when required.

The system also includes a touchscreen controller that allows real-time monitoring and management of system parameters. This controller communicates with the system via digital input/output (DI/DO) connections, allowing for smooth operation and coordination of all components involved in the disinfection process.

### 3.4. Simulation Model

**Figure 6:**
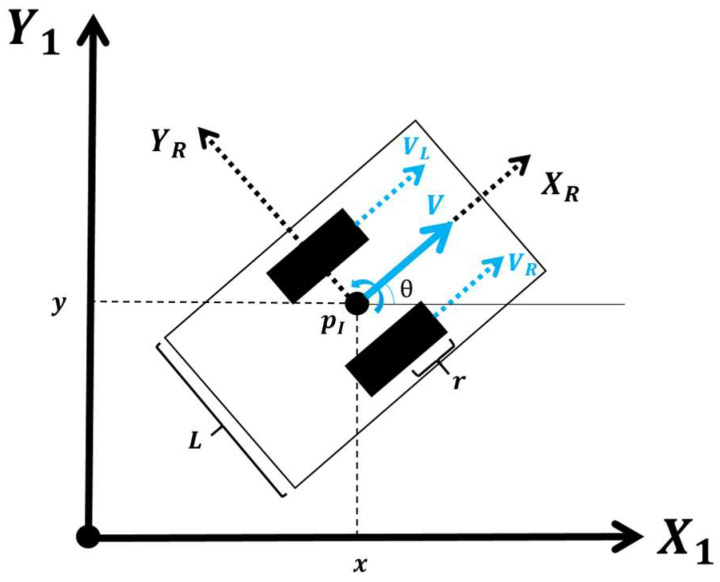
Kinematic model of the Hussar robot [38].

In order to optimize the trajectories, it is necessary to have a simulation model to replicate the behavior of the robot and the lamp. A simulation environment of the Hussar robot has been created to replicate its movement within defined spaces. This setup required defining the type of robot, modeling its motion dynamics, and setting the relevant control parameters. The kinematic model of the Hussar robot is illustrated in Figure 6 and is expressed mathematically in Equations (2)–(4). This model was adapted from [41].

In this scheme, the position of the robot is defined by its Cartesian coordinates $p_{I}=\{x,y,\theta \}$ within the inertial frame $\left\{X_{I},Y_{I}\right\}$. The transverse velocity $Y_{R}$ is assumed to be zero, as there is no lateral slip.

The longitudinal velocity V and angular speed W are determined using the linear velocities of each wheel, $V_{R}$ and $V_{L}$, as in Equation (2), where the subindexes *R* and *L* denote the right and left wheel, respectively.

(2) $$V=\frac{V_{R}+V_{L}}{2},\;\;\;\;\;\;\;\;\;\;\;W=\frac{V_{R}-V_{L}}{L}$$

The left and right wheel speeds are regulated by a controller so as to follow a specified longitudinal and angular velocity profile, which can be modeled using Equations (3) and (4).

(3) $$\left\{\begin{array}{c} V\left(t\right)=V_{0}+\alpha_{V}\left(t-t_{0}\right)\;\;\;\\ V\left(t\right)=V_{ref}\;\;\;\;\;\;\;\;\;\;\;\;\;\;\;\;\;\;\;\;\;\;\;\;\;\; \end{array}\right.\;\;\;\;\;\;\;\;\;\;\;\;\;\;\begin{array}{c} V<V_{ref}\\ V=V_{ref} \end{array}$$

(4) $$\left\{\begin{array}{c} W\left(t\right)=W_{0}+\alpha_{W}\left(t-t_{0}\right)\;\;\;\\ W\left(t\right)=W_{ref}\;\;\;\;\;\;\;\;\;\;\;\;\;\;\;\;\;\;\;\;\;\;\;\;\;\;\; \end{array}\right.\;\;\;\;\;\;\;\;\;\;\;\begin{array}{c} W<W_{ref}\\ W=W_{ref} \end{array}$$

The speed profile (both longitudinal and angular) is assumed to have a trapezoidal shape. Longitudinal acceleration is represented by $\alpha_{V}$ and the angular acceleration is denoted by $\alpha_{W}$. These acceleration values differ for the phases of acceleration and deceleration, allowing the speed to increase or decrease linearly until it reaches the target speeds: $V_{ref}$ for longitudinal and $W_{ref}$ for angular speed.

**Figure 7:**
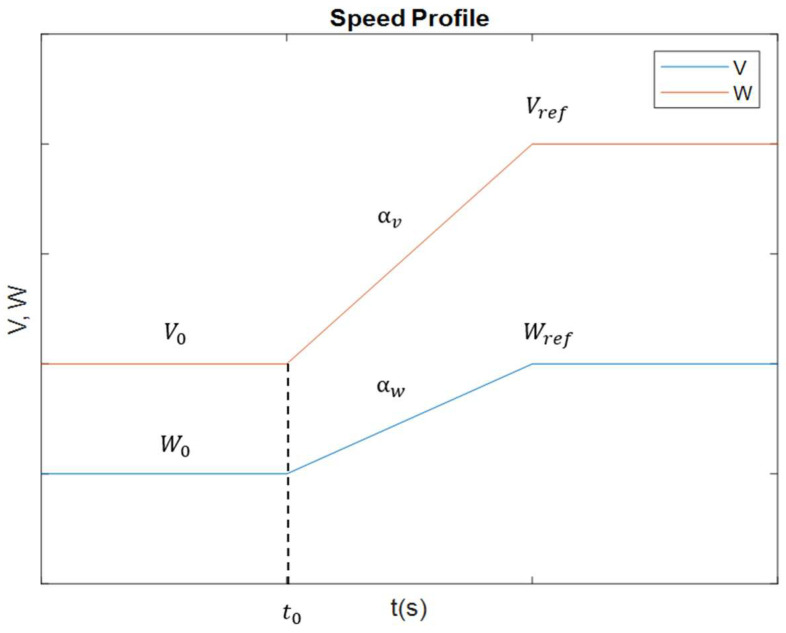
Speed profiles of the robot (red: longitudinal; blue: angular) [40].

The velocity profiles defined by Equations (4) and (5) are illustrated in Figure 7, where the red line represents the longitudinal speed and the blue line the angular speed.

It is worth mentioning that this approach to implementing velocity profiles is common in mobile robotics, although other profiles could also be applied, simply by adjusting the acceleration and maximum velocity parameters (both longitudinal and angular).

Assuming that $p_{I}$ is an arbitrary position in the global inertia frame, the kinematic model is given by Equation (5).

(5) $${\dot{p}}_{I}=\left[\begin{array}{c} \dot{x}\\ \dot{y}\\ \dot{\theta } \end{array}\right]=\left(\begin{array}{ccc} \cos\left(\theta \right) & -\sin\left(\theta \right) & 0\\ \sin\left(\theta \right) & \cos\left(\theta \right) & 0\\ 0 & 0 & 1 \end{array}\right)\times \left[\begin{array}{c} V\\ 0\\ W \end{array}\right]$$

The state vector is defined by [x, y, θ], where all Cartesian coordinates are measured in m, angles in rad, linear velocities in m/s, and angular velocities in rad/s.

The radiation matrix, which represents the radiation at each point of the virtual environment where the robot operates, is calculated using Equation (6) [38].

(6) $$R_{M}(i,j,t)=P_{L}\cdot k\int \frac{1}{D_{i,j}^{2}(t)}dt$$

**Figure 8:**
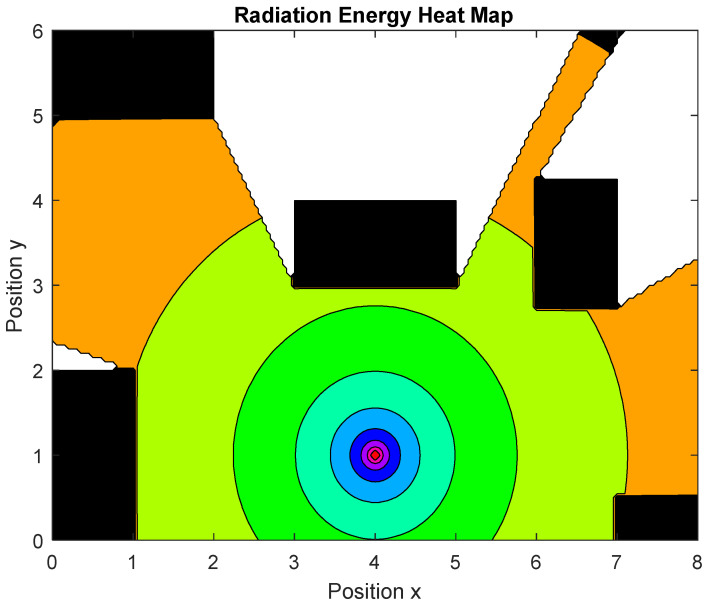
Radiation beam simulation. Black rectangles are obstacles. White color indicates no radiation. All other colors indicate radiation.

The disinfection system, modeled as a 207J high-energy pulse lamp, has been simulated by projecting sequential straight lines, each representing a beam of light emitted from the lamp location at 5-degree intervals and a spread of 10 m. It has been assumed that beyond 10 m, the radiation can be considered negligible. The intersection of each beam with obstacles is calculated by segmenting the beam at the moment of collision, allowing the system to exclude any radiation blocked by opaque objects in the environment. This approach ensures that shaded areas are ignored by the program, providing accurate calculations of exposed surfaces (Figure 8).

## 4. Optimization Methodology

**Figure 9:**
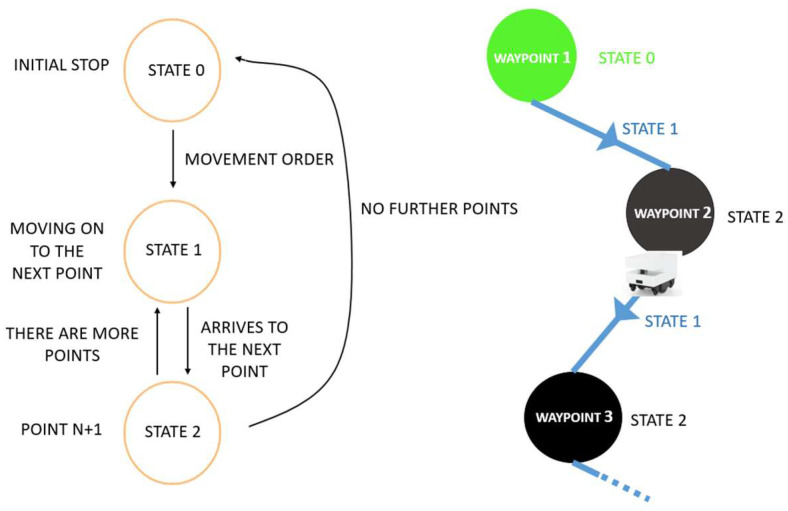
State diagram; (**right**) state machine; and (**left**) states [41].

To determine the most efficient path within a designated area, allowing the autonomous robot to UVC disinfect that space, specific waypoints are defined to trace the disinfection path. The robot is programmed to recognize a fixed sequence of coordinates and move through them in a predetermined order. Its motion is governed by a state machine, shown in Figure 9 (left), and Figure 9 (right) shows the states reached along its path. This approach is followed by the robot path simulation (offline process in Figure 4) and by the Raspberry to send the positions to the Hussar robot (online process in Figure 4).

Each waypoint is defined by the following four parameters: the coordinates (*x*, *y*), the angle θ, and the stop time t; that is, $\left(x_{r},y_{r},\theta_{r},\;t_{r}\right)$, where the sub-index r indicates the robot. To avoid excessive program execution time, the angles have been set to fixed values. Since the disinfection area is circular (Figure 8), these angles do not affect the trajectory and mainly influence the computational load. By keeping the angles constant, the algorithm control variables are limited to the position coordinates ($x_{r},y_{r}$) only.

**Figure 10:**
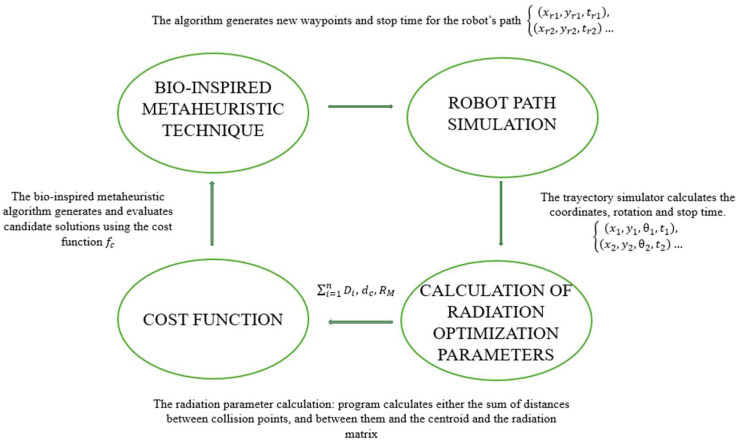
Optimization methodology.

The optimization methodology is divided into four main modules that determine the efficient route to ensure sufficient radiation coverage for disinfection (Figure 10).

The bio-inspired metaheuristic technique generates waypoints and determines the required stopping times at each point of the robot’s trajectory. By using a bio-inspired metaheuristic approach, such as the PSO, GOA, WOA or BA, the waypoints are iteratively refined to improve coverage. The output of this module is a set of waypoints and stopping times, represented as $\left(x_{r1},y_{r1},t_{r1}\right),\left(x_{r2},y_{r2},t_{r2}\right)$…

In the trajectory simulation module, the system evaluates the robot’s trajectory based on the proposed waypoints. The simulator calculates the robot’s coordinates, orientation, and stopping points. That is, the robot’s motion along each position is simulated, generating the coordinates $\left(x_{1},y_{1},\theta_{1},t_{1}\right),\;\left(x_{2},y_{2},\theta_{2},t_{2}\right)$…, which reflects the robot’s actual trajectory.

After simulating the path, the radiation parameters are calculated. This module calculates the cumulative radiation coverage based on the distance between points $R_{M}$, the distances to collision points $\sum_{i=1}^{n}D_{i}$, and the robot’s position relative to the centroid and the radiation matrix $d_{c}$.

The cost function uses the parameters from the previous steps to evaluate the effectiveness of the proposed route. This cost function $f_{c}$ considers the total distance traveled, the distribution of waypoints in the quadrants of the area, and the level of radiation coverage relative to a given threshold value.

### Optimization Problem

To ensure the correct and sufficient radiation dose at all points within the space to be disinfected, the cost function described in (7) is used.

(7) $$f_{c}=\left\{\begin{array}{cc} 3+\sum_{i=1}^{n}D_{i} & if\;CC\\ 2+\left(1-\frac{\bar{d_{centroid}}}{Max\left(d_{centroid}\right)}\right) & if\;NCC-NDP\\ 1+\frac{\sum_{i=1}^{n}{∆R}_{i}}{thr\cdot n_{cells}} & if\;NCC-DPBT\\ \frac{1}{N}\cdot \sum_{i=1}^{N}\left|Obj_{rad\left(i\right)}-real_{rad\left(i\right)}\right| & \;\;\;\;\;\;\;\;\;\;\;\;\;\;\;\;\;\;if\;NCC-DPAT \end{array}\right.$$

This cost function operates in a sequential manner. As the algorithm discovers better solutions, it progresses through different stages of the function, with each improvement leading to a lower cost-function value. These stages are organized into levels to clearly indicate the value of the cost function at each level. The different conditions are shown in Equations (8)–(11). In these equations CC, NCC−NDP, NCC−DPBT, and NCC−DPAT refers to the “collision condition”, “non collision condition with no distributed path”, “non collision condition with distributed path and radiation below threshold”, and “non collision condition with no distributed path and radiation above threshold”, respectively.

(8) $$CC:\sum_{i=1}^{n}D_{i}\neq 0$$

(9) $$NCC-NDP:\sum_{i=1}^{n}D_{i}\neq 0\wedge ∄\left(x_{i},y_{i}\right)\in Q_{1-4}$$

(10) $$NCC-DPBT:\sum_{i=1}^{n}D_{i}\neq 0\wedge \exists \left(x_{i},y_{i}\right)\in Q_{1-4}\wedge \exists \left(i,j\right){:R}_{M}<thr$$

(11) $$NCC-DPAT:\sum_{i=1}^{n}D_{i}\neq 0\wedge \exists \left(x_{i},y_{i}\right)\in Q_{1-4}\wedge ∄\left(i,j\right){:R}_{M}<thr$$

The first stage of the cost function is defined for trajectories that result in collisions with obstacles in the environment. When this occurs, the cost function takes a value greater than 3, calculated as 3 plus the sum of the distances crossed with various obstacles. At this stage, n is the number of obstacles and $D_{i}$ is the distance that the vehicle’s trajectory crosses obstacle i.

After this initial stage, when the algorithm identifies collision-free trajectories, the cost function evaluates the compactness of the route by considering the distance to the centroid (12).

(12) $$d_{centroid}=\sqrt{{\left(x_{i}-\bar{x}\right)}^{2}+{\left(y_{i}-\bar{y}\right)}^{2}}\;$$

**Figure 11:**
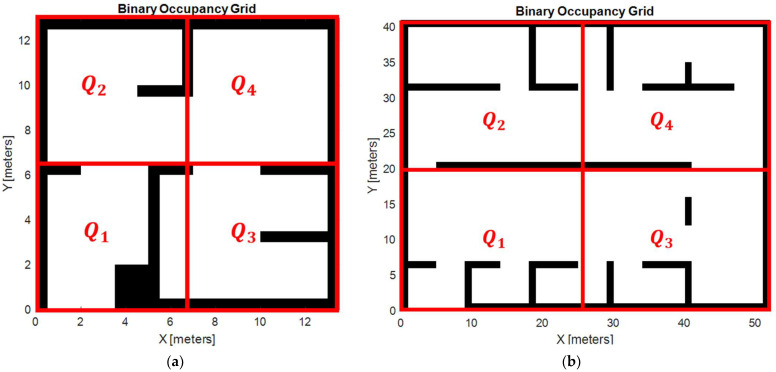
Quadrants for less complex (**a**) and highly complex (**b**) environments.

The centroid represents the geometric center of the set of waypoints along the route, so the distance to centroid metric makes the algorithm find more centralized and compact routes. At this stage, the cost function penalizes trajectories where waypoints are too clustered, promoting routes that are more evenly distributed along the disinfection area. Specifically, this stage of the cost function is triggered when there is fewer than one waypoint in each quadrant $Q_{1-4}$, ensuring balanced coverage across the space (Figure 11a,b). The goal is to avoid excessive concentration in certain areas, which could lead to local minima that would make coverage redundant and travel time inefficient.

The third phase of the cost function addresses the adequacy of radiation coverage by penalizing areas where radiation levels fall below the required threshold. The expression $\frac{\sum_{i=1}^{n}{∆R}_{i}}{thr\cdot n_{cells}}$ calculates this penalty, where the numerator represents the cumulative difference between the threshold radiation level and the actual radiation for each cell ($\sum_{i=1}^{n}{∆R}_{i}$). The denominator normalizes this penalty by multiplying the threshold radiation level (thr) by the total number of cells in the area, $n_{cells}$.

In the last phase, the mean absolute error (MAE) of the matrix $R_{M}$ is calculated. That is, it considers the absolute difference between the actual real_rad and the target radiation, Obj_rad.

## 5. Bio-Inspired Metaheuristic Techniques Selected

In this work, the following four bio-inspired algorithms have been compared to optimize AGV navigation in complex environments: bat algorithm (BA), whale optimization algorithm (WOA), gazelle optimization algorithm (GOA), and particle swarm optimization (PSO). Each of these algorithms is briefly described in the following subsections.

### 5.1. Bat Algorithm (BA)

The bat algorithm (BA) is inspired by the echolocation behavior of bats. In BA, each bat represents a solution within the search space, and its position is iteratively updated based on the bat’s current speed and the best solution found so far. Bats emit sound pulses, and the frequency of these pulses plays a fundamental role in their search behavior, where higher frequencies lead to faster exploration of the space and lower frequencies allow for a more focused local search. The position of each bat is updated as follows:

(13) $$x_{i}^{t+1}=x_{i}^{t}+v_{i}^{t+1}$$

In (13), $x_{i}^{t+1}$ is the bat position at iteration t+1; $x_{i}^{t}$ is the bat position at iteration t; and $v_{i}^{t+1}$ represents the updated velocity, which is influenced by the difference between the current position and the best position known. This movement system allows bats to navigate through the search space by adjusting both their movement frequency and sound level, which represents how “eager” they are to explore. As the algorithm progresses, the volume decreases as bats approach promising solutions, focusing their efforts on exploiting local optima. Furthermore, the pulse emission rate controls the balance between exploration and exploitation, where higher rates correspond to a wider exploration of the search space.

### 5.2. Whale Optimization Algorithm (WOA)

The whale optimization algorithm (WOA), inspired by the hunting strategies of humpback whales, mimics their spiral-shaped bubble-net behavior to trap preys. In the WOA, solutions, or whales, move through the search space by alternating between two modes: a shrinking circle around the current best solution and a spiral movement toward the prey, which represents the optimal solution. The whale’s position at each iteration is updated by a combination of the best position known and a random adjustment that mimics the whale’s circular or spiral motion.

The shrinking encircling behavior can be described as an update of the position that brings the whale closer to the best solution, the prey. The encircling phase can be represented by the following equation:

(14) $$\vec{X}\left(t+1\right)=\vec{X^{*}}\left(t\right)-A\cdot \left|\vec{C}\cdot \vec{X^{*}}\left(t\right)-\vec{X}\left(t\right)\right|$$

where $\vec{X^{*}}\left(t\right)$ is the best solution found, A and C are random vectors, and $\vec{X}\left(t\right)$ is the position of a whale at iteration t.

The spiral path introduces stochastic elements into the search, helping to prevent premature convergence and fostering exploration. The spiral updating phase is represented by the following equation:

(15) $$X\left(t+1\right)=\left|\vec{X^{*}}\left(t\right)-\vec{X}\left(t\right)\right|\cdot e^{bl}\cdot \cos\left(2\pi l\right)+\vec{X^{*}}\left(t\right)$$

where l is a random number in [−1, 1]; and b defines the shape of the spiral.

This dual mechanism of exploration and exploitation allows the WOA to efficiently search large and complex solution spaces.

### 5.3. Gazelle Optimization Algorithm (GOA)

The gazelle optimization algorithm (GOA) models the evasive and unpredictable movements of gazelles when fleeing from predators and uses this behavior to guide the search for optimal solutions. In the GOA, each gazelle’s position update can be described as a combination of its previous position, random factors representing sudden evasive movements, and a gradient component guiding it toward better solutions, as follows:

(16) $$\vec{X_{i}}\left(t+1\right)=\vec{X_{i}}\left(t\right)+r_{i}\left(t\right)\cdot \vec{v_{i}}\left(t\right)+s_{i}\left(t\right)\cdot \nabla f\left(\vec{X_{i}}\left(t\right)\right)$$

where $r_{i}\left(t\right)$ is a random factor, $\vec{v_{i}}\left(t\right)$ is the velocity of the gazelle, and ∇f is the gradient of the objective function.

The gazelle’s ability to sense its environment allows it to assess the search space and adapt its movements dynamically. As the algorithm progresses, the gazelle population increasingly focuses on promising regions of the search space, while random, one-off changes prevent them from becoming trapped in local optima. The GOA also incorporates a mutation mechanism that simulates sudden changes in direction when gazelles evade predators. This stochastic element ensures diversity in the population, further improving the algorithm’s ability to explore the search space.

### 5.4. Particle Swarm Optimization (PSO)

The particle swarm optimization (PSO) algorithm is inspired by the collective behavior of flocks of birds or schools of fish. Each particle ($x_{i}^{t}$) represents a possible solution in the search space. The position of each particle is updated based on its own best experience and the best-known solution found by the swarm. This position update can be expressed by the following equation:

(17) $$x_{i}^{t+1}=x_{i}^{t}+v_{i}^{t+1}$$

where the updated velocity ($v_{i}^{t+1}$) is determined by both the particle’s own best position and the global best position, following the equation:

(18) $$v_{i}^{t+1}=\omega v_{i}^{t}+c_{1}r_{1}\left(p_{i}^{t}-x_{i}^{t}\right)+c_{2}r_{2}\left(g^{t}-x_{i}^{t}\right)$$

In this equation, inertia (ω) controls the impact of previous velocities ($v_{i}^{t}$), while cognitive and social terms ($c_{1}$ and $c_{2}$) determine how much influence the particle’s best position ($p_{i}^{t}$) and the swarm’s best global position ($g^{t}$) have on its velocity. $r_{1}$ and $r_{2}$ are random factors between 0 and 1 ($r_{1},\;r_{2}\;$∈ [0, 1]) that introduce stochasticity to allow a broader exploration of the search space.

PSO is very efficient due to its balance between exploration (through randomization and velocity updates) and exploitation (convergence towards the best particular and global positions).

## 6. Results

### 6.1. Simulation Scenarios

The robot path optimization has been simulated in two distinct scenarios, both modeled as occupancy grids generated using the MATLAB 2021b software. These grids represent areas with obstacles, providing a realistic testbed to evaluate the robot’s navigation and disinfection capabilities.

**Figure 12:**
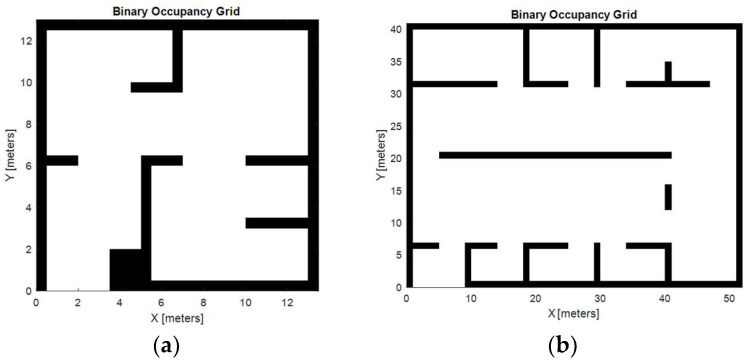
Simulation scenarios: low complexity (**a**) and high complexity (**b**) [38].

The first environment, depicted in Figure 12a, is a simple complexity map containing 12 obstacles. The grid resolution for this environment is 0.5 m per cell, allowing fine granularity for robot positioning and obstacle avoidance. This map represents a compact environment with narrower passages and confined spaces, to simulate scenarios such as offices, small hospital rooms, or hallways. It presents a challenge for the robot because it requires precise movement and good path planning to avoid collisions while ensuring complete coverage.

The second scenario, depicted in Figure 12b, is a larger and more complex map. Certain obstacles were removed for the simulation to make the problem computationally feasible while maintaining a high level of complexity. Specifically, the map, which originally contained 33 obstacles, was simplified to 20 for this simulation. The grid resolution in this case is 1 m per cell, which is suitable for larger, open environments. This map represents areas such as warehouses, large hospital wings, or public spaces, which allow for easier navigation, although the robot must ensure complete coverage of the environment.

In both environments, the robot navigates completely autonomously, using the occupancy grid to plan its route, avoid obstacles, and reach all the designated disinfection points. The difference in obstacle density and map size between the two scenarios provides valuable insights into how the robot adapts its path planning to different environments, optimizing its movement and energy usage for effective disinfection.

### 6.2. Evaluation Metrics

For each of the scenarios the optimization algorithms were implemented using MATLAB, and their performance was evaluated based on the following metrics:

- Non-collision Time With Non-uniform Path: time required for the algorithm to ensure the robot avoids obstacles in a non-uniformly distributed path;
- Non-collision Iteration With Non-uniform Path: number of iterations required for the algorithm to ensure the robot avoids obstacles in a non-uniformly distributed path;
- Non-collision Time With Distributed Path (Below Threshold Radiation): time taken for the robot to avoid collisions while following a uniformly distributed path with areas of radiation below the threshold;
- Non-collision Iteration With Distributed Path (Below Threshold Radiation): number of iterations it takes to ensure the robot avoids collisions while following a uniformly distributed path with areas of radiation below the threshold;
- Non-collision Time With Distributed Path (Above Threshold Radiation): time taken to achieve a collision-free, uniformly distributed path that meets the radiation threshold across the entire space;
- Non-collision Iteration With Distributed Path (Above Threshold Radiation): number of iterations it takes to achieve a collision-free, uniformly distributed path that meets the radiation threshold across the entire space;
- Optimal Solution Time: the minimum time required to reach the optimal solution;
- Iterations: number of iterations it takes to reach the optimal solution;
- Optimal Path Time: the shortest trajectory time achieved in the optimal solution;
- Optimal Path Distance: the path distance achieved in the optimal solution;
- Optimal Path Coverage: the percentage of the area that has received at least 1% of the target radiation;
- Optimal Path Disinfection: The total disinfection achieved in the optimal solution. It is assumed that all cells in the map are equally infected before the disinfection process begins;
- Maximum Radiation per Cell: the maximum radiation value recorded per cell in optimal solution.

The first four metrics refer to obstacle avoidance in the different scenarios. These metrics measure how quickly the algorithms can ensure safe navigation, either on a non-distributed path or on a distributed path where the radiation falls below the required threshold. The fifth and sixth metrics evaluate the speed at which each algorithm reaches a feasible solution. The seventh and eighth metrics evaluate the efficiency of the path in terms of distance and time of the optimized path. Finally, the ninth and tenth metrics capture the efficiency of the path in terms of distance and time on the optimized path.

The GOA, WOA, BA, and PSO algorithms require an initial trajectory to begin the optimization process. In the low-complexity scenario, a rectangular trajectory with a random offset of up to 2 m was used, providing a simple but varied baseline for each algorithm. For the high-complexity scenario, an initial trajectory was implemented that traverses all rooms in the environment to speed up the search process, but deliberately includes a collision point to test the algorithms’ ability to effectively detect and resolve collisions. This approach ensures that the initial conditions are sufficiently challenging to assess each algorithm’s ability to refine the trajectory and achieve an optimal, collision-free trajectory.

For both scenarios, less and more complex, the results are organized as follows: First, the different metrics for the simple scenario, and the evolution of the cost function for each algorithm in this setting will be presented in tables. Next, the trajectory improvements achieved by each algorithm are then shown, highlighting the initial and optimized trajectories in each case. Finally, a 3D graph illustrates the radiation exposure through space for each optimal trajectory.

All simulations were carried out on a Dell Vostro 5471 computer, equipped with eight GB of RAM and an eighth generation Intel i7 processor, using MATLAB version 2021b.

### 6.3. Results in Low-Complexity Scenario

Table 1 provides the computational time required by each optimization algorithm, in a low-complexity setting, to find obstacle-free paths that meet the distributed radiation thresholds. From this table, one can observe the time required by each algorithm to achieve a spatially distributed, collision-free solution (No Coll. Time (NUP)); a spatially distributed but collision-free solution below the radiation threshold (No Coll. Time (DP-BT)); and a spatially distributed, collision-free solution that meets the threshold required to deactivate SARS-CoV-2 (No Coll. Time (DP-AT)), as well as the time required to find the optimal solution (Opt. Sol. Time).

The results indicate that all algorithms perform similarly in terms of computation time. The bat algorithm (BA) stands out for finding an initial solution quickly. However, it fails to improve this solution in the 3 h of the experiment. This is also shown in Figure 9 (Cost Function), where BA produces the least optimal trajectory. This highlights its limitations in refining solutions over long computation times.

Table 2 shows the results in terms of the number of iterations. The bat algorithm (BA) stands out again, achieving the fewest iterations in most metrics, reflecting its fast convergence in low-complexity settings. The whale optimization algorithm (WOA) requires a moderate number of iterations and performs reasonably well in scenarios with collision-free distributed paths. In contrast, the particle swarm optimization (PSO) requires significantly more iterations, especially to achieve distributed paths with adequate radiation levels, corresponding to a higher overall computational time.

**Figure 13:**
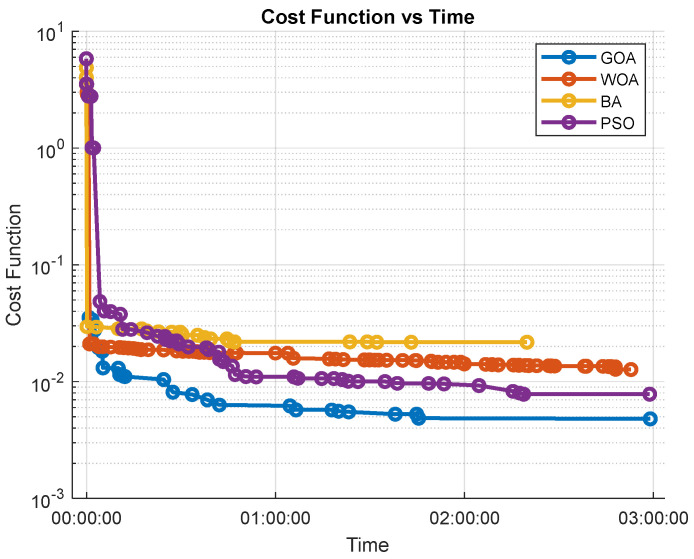
Simple environment cost function evolution.

This experimentation in a low-complexity setting highlights the relative efficiency of each algorithm when the optimal solution is close to the initial configuration. The BA’s ability to quickly adapt to the environment and meet constraints makes it a solid choice for simple configurations when a fast solution is desired, as demonstrated by its good performance in both time- and iteration-based metrics. On the other hand, while PSO and GOA are noticeably slower, Figure 13 reveals that they achieve better solutions. In other words, their refinement capacity, even with higher computational times and iteration counts, results in more optimal paths, making them highly suitable when quality is prioritized over speed in simple settings.

The final results for each optimized trajectory are detailed in Table 3. This table presents the shortest trajectory time achieved in the optimal solution (Opt. Path Time), the total distance traveled by the best solution (Opt. Path Distance), the percentage of the area that has received at least 1% of the target radiation (Opt. Path Coverage), the total disinfection achieved (Opt. Path Disinfection), and the maximum radiation value recorded in the map (Max Radiation). The results demonstrate that all algorithms provide acceptable solutions in terms of coverage and disinfection. Notably, the two algorithms with the best overall performance also maintain lower levels of maximum radiation.

Figure 13 shows the evolution of the cost functions for the four bio-inspired algorithms. In this graph it can be observed that all the techniques converge at a similar speed, with the GOA and PSO achieving a slightly better solution in simpler environments.

**Figure 14:**
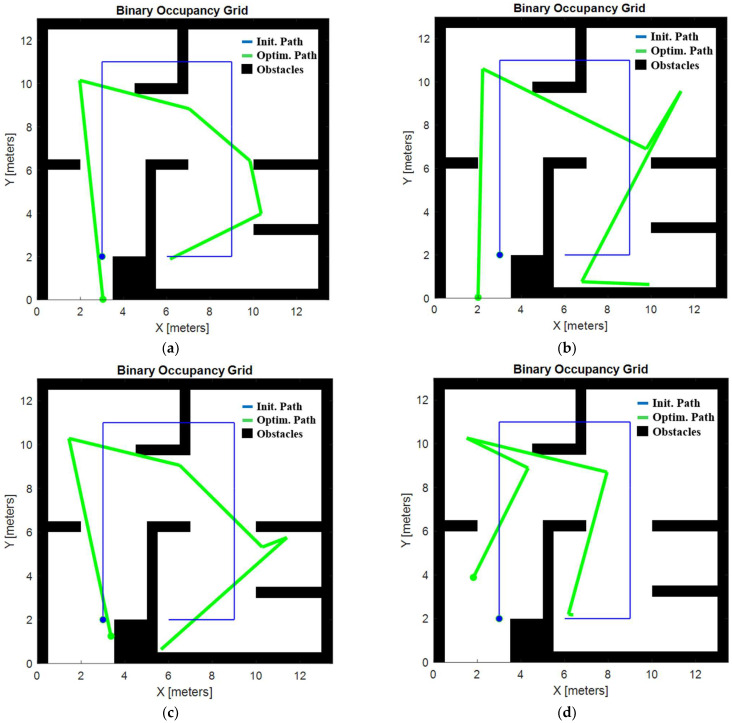
Low-complexity environment. Initial and optimized trajectory with the GOA (**a**), initial and optimized trajectory with the WOA (**b**), initial and optimized trajectory with the BA (**c**), and initial and optimized trajectory with the PSO (**d**).

Figure 14a–d show the optimal trajectories obtained with each algorithm. While all these trajectories are feasible and valid, the two shown in Figure 14a,d stand out in terms of efficiency. These trajectories, unlike the others, lack sharp turns and unnecessary backtracking to previously covered areas, which reduces radiation overexposure in already treated spaces. This reduction in redundant coverage results in a lower cost function, making these trajectories more optimal. These trajectories were achieved with both the GOA and PSO algorithms. In particular, the trajectory produced by the GOA provides the least amount of radiation overexposure, positioning it as the best option among all the trajectories tested and establishing the GOA as the most effective algorithm for optimizing radiation trajectories in simple environments.

**Figure 15:**
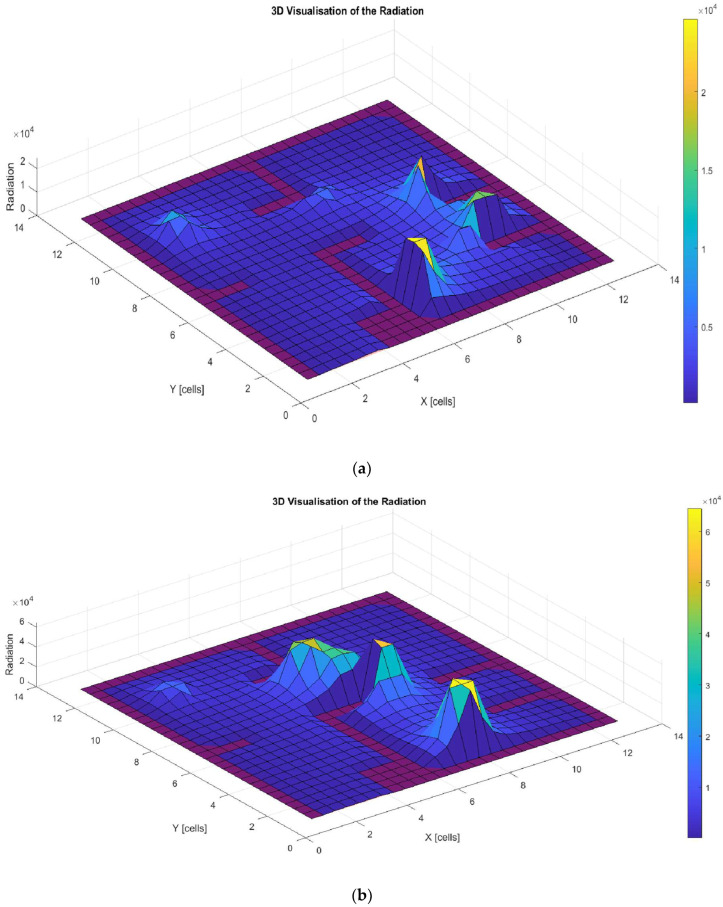

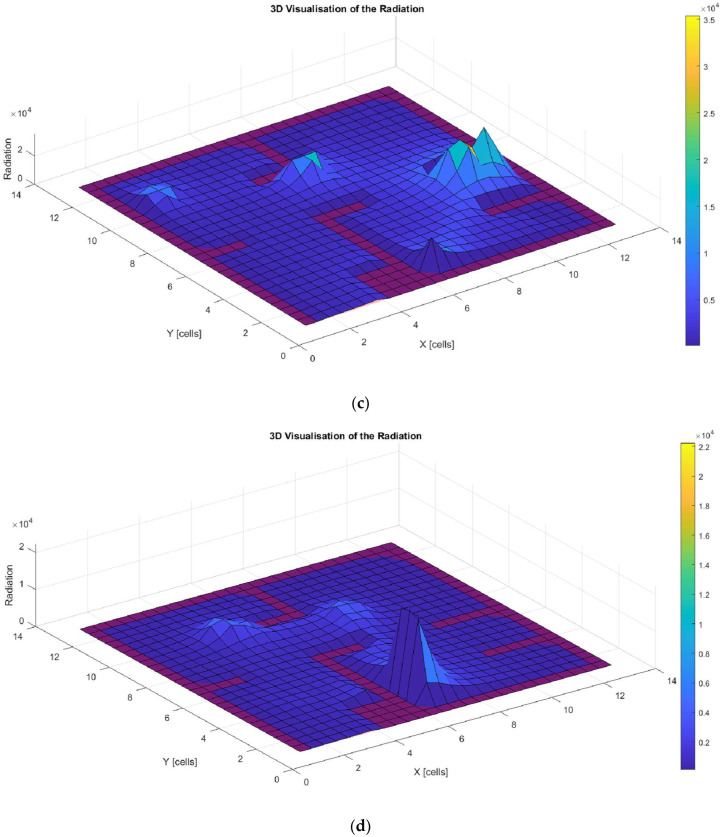
Low-complexity environment. Three-dimensionalradiation with the GOA (**a**), 3D radiation with the WOA (**b**), 3D radiation with the BA (**c**), and 3D radiation with the PSO (**d**).

Finally, Figure 15a–d illustrate the amount of radiation generated along the trajectories shown in Figure 14a–d. These graphs show that all solutions exceed the proposed radiation threshold (500 J/m^2^), with noticeable peaks at the points where the robot stops and turns. This is due to the increased exposure time at these specific locations.

### 6.4. Results in High-Complexity Scenario

Table 4 shows the performance of each optimization algorithm in a high-complexity setting, evaluating their computational efficiency and ability to generate collision-free trajectories that meet the distributed radiation thresholds. This table details the computational time required by each algorithm to produce a spatially distributed, collision-free solution (Non-Collision Time (NUP)); a sub-threshold distributed trajectory (Non-Collision time (DP-BT)); and a spatially distributed collision-free solution that meets the radiation threshold needed for SARS-CoV-2 deactivation (Non-Collision Time (DP-AT)), as well as the optimal solution (Optimal Sol. Time).

In this more challenging scenario, the bat algorithm (BA) continues to show competitive performance, achieving fast collision-free times and effectively progressing towards the optimal solution. The particle swarm optimization (PSO) algorithm also shows stable performance, balancing its speed with high-quality trajectory results. However, the whale optimization algorithm (WOA) has many difficulties in this high-complexity setting and fails to produce a collision-free distributed solution. This limitation can be attributed to the WOA’s tendency to settle into a local minimum, which prevents it from fully optimizing the trajectory.

These results highlight the limitations of the WOA in more complex spaces, where its search capability may be hampered by local optima. In contrast, the GOA and PSO algorithms demonstrate greater adaptability in refining trajectories in a high-complexity setting, although GOA takes a noticeably longer time to find initial collision-free solutions.

In Table 5 we can see that the GOA requires a larger number of iterations than the others, reflecting its slow but thorough search process, ultimately achieving the shortest optimal path distance. The BA also demonstrates good performance, finding a collision-free solution relatively quickly and steadily progressing towards an optimal path, although it results in a slightly longer path distance compared to the GOA. The particle swarm optimization (PSO) works efficiently, with few iterations to achieve collision-free solutions, but its final path distance is longer than that of GOA.

As well as in the simple scenario, the outcomes of each optimized trajectory for the complex environment are summarized in Table 6. This table also includes the minimum time required to complete the optimal trajectory (Opt. Path Time), the total path length covered in the best solution (Opt. Path Distance), the percentage of the area that has received at least 1% of the target radiation (Opt. Path Coverage), the overall disinfection performance (Opt. Path Disinfection), and the highest radiation value observed in the map (Max Radiation). In this table, it is evident that the algorithms with the best final cost functions also exhibit lower maximum radiation levels. Additionally, the WOA, having been trapped in a local minimum, failed to reach all cells and was unable to achieve the target radiation threshold in them.

**Figure 16:**
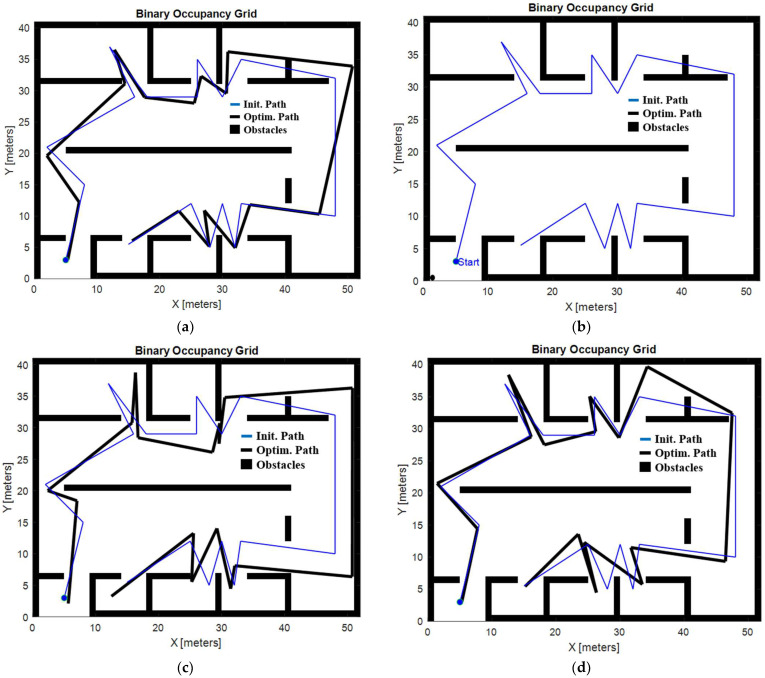
High-complexity environment. Initial and optimized trajectory with the GOA (**a**), initial and optimized trajectory with the WOA (**b**), initial and optimized trajectory with the BA (**c**), and initial and optimized trajectory with the PSO (**d**).

Figure 16 shows the optimized trajectories generated by each method. In particular, it can be observed that the WOA method produced a trajectory that is concentrated around a local minimum. Although this trajectory successfully avoids collisions, it fails to adequately explore the entire available space, which limits its effectiveness in covering the entire area. This is a limitation of the WOA compared to the other methods that achieve more complete spatial coverage.

**Figure 17:**
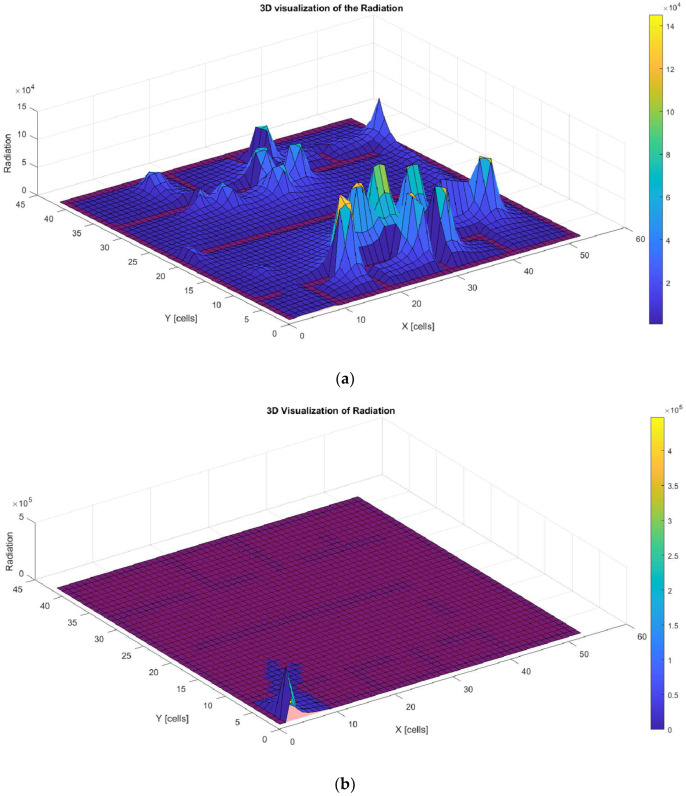

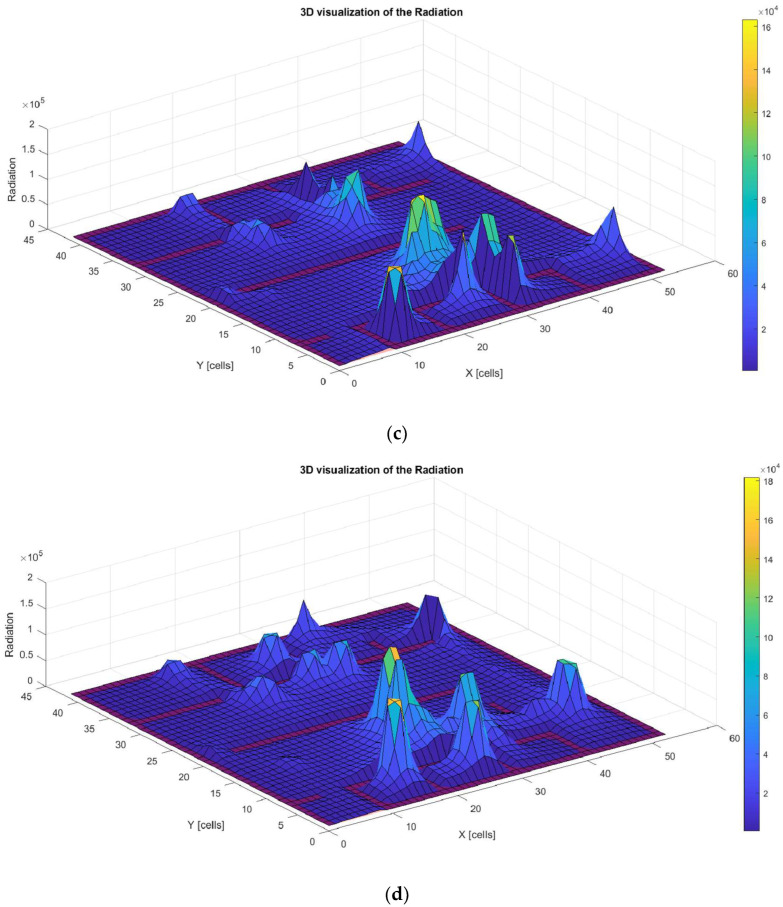
High-complexity environment. Three-dimensionalradiation with the GOA (**a**), 3D radiation with the WOA (**b**), 3D radiation with the BA (**c**), and 3D radiation with the PSO (**d**).

In the complex environment, the optimized trajectories generated by each method reveal remarkable similarities regarding coverage. The highest peaks of radiation are located at points where the robot stops or changes direction. These peaks could be minimized by reducing the stopping time, an improvement that could be achieved with an extended runtime for bio-inspired metaheuristic techniques. Furthermore, the WOA shows limitations by concentrating around a local minimum, leaving portions of the space unreached, although it effectively avoids obstacles (Figure 17).

**Figure 18:**
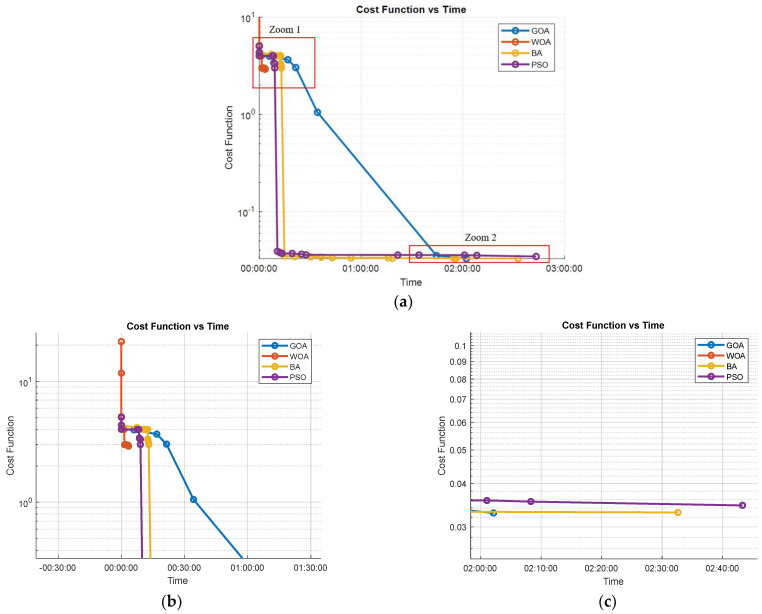
Complex environment cost functions (**a**), Zoom 1 area (**b**) and Zoom 2 area (**c**).

Finally, Figure 18a illustrates the variation in the cost functions of each method over time. As can be seen, the cost function of WOA gets stuck at a local minimum, while the other methods achieve reasonably good values that ensure complete radiation above the threshold required to disable COVID-19. This limitation of WOA may be related to the design of the algorithm, which mimics the behavior of whales following a leader during hunting. Unlike the other algorithms, WOA lacks any mechanism to escape from local minima. Figure 18b,c provide a more detailed view of the final cost function values shown in Figure 18a for a better comparison.

At the end of the experiment, the final cost function values for each algorithm describe their performance in this complex environment. The GOA achieved the lowest final cost function value, 0.00328, indicating a highly efficient route with optimal radiation coverage. The BA followed closely with a final cost of 0.0033, also achieving complete and effective coverage but slightly less refined than the GOA. The PSO ended with a cost function value of 0.00346, reflecting a good solution that ensures adequate coverage but with minor path inefficiencies. In contrast, the WOA’s final cost function value was significantly higher at 2.917, confirming its tendency to get trapped in local minima without achieving the optimal path required for comprehensive disinfection.

These final values further underline the strengths of GOA and BA in complex environments, while highlighting the limitations of WOA in such scenarios due to its lack of adaptability in high-iteration contexts.

## 7. Conclusions

In this work, four meta-heuristic strategies (GOA, WOA, BA, and PSO) are applied and compared for the optimization of a CCPP problem, where a mobile robot equipped with a UVC lamp performs an automatic disinfection task on a surface.

This study shows that the gazelle algorithm consistently outperforms three other meta-heuristic algorithms in optimizing UVC disinfection routes, both in simple and complex environments. Its adaptability and resistance to premature convergence allow it to efficiently explore and exploit the search space, generating optimized trajectories that guarantee comprehensive coverage, minimize travel time, and reduce radiation exposure. This versatility makes this algorithm particularly effective for autonomous disinfection in a wide range of spaces, both dense in obstacles and wide, open areas. It could be applied to any critical infrastructure (hospitals, airports, train stations, shopping malls, etc.). Furthermore, its robust performance facilitates its applications to industrial environments, such as manufacturing plants, or smaller facilities such as offices or classrooms.

In complex environments, the bat algorithm also showed promising performance, along with particle swarm optimization. Both the BA’s echolocation-inspired and the PSO’s collective behavior-inspired mechanisms excel at navigating intricate spaces, making them particularly useful in environments with numerous obstacles or partitions. For example, the bat algorithm could be applied inside industrial plants, where machinery and structure create a complex layout, or in office buildings with dense cubicle arrangements. However, its tendency to stall after initial convergence limits its effectiveness in operations that require lengthy optimization, such as nightly disinfection routines in large facilities.

However, the whale optimization algorithm showed notable difficulties in complex environments. The algorithm’s reliance on a spiral motion pattern, inspired by whaling eddies, makes it prone to getting stuck in local minima. This feature, while potentially advantageous for full exploration in simpler scenarios, limits its effectiveness in highly complex environments where continuous refinement and flexibility are essential to finding optimal solutions. In this sense, the whale optimization algorithm could be suitable for applications in open, low-convolution spaces, such as warehouses or parking lots, where the absence of many obstacles reduces the need for advanced route refinement. Its simplicity could also be leveraged in small facilities where sanitization requirements are less demanding.

Overall, these findings highlight the superior performance of the GOA in a variety of scenarios, making it the most robust choice for real-world applications requiring precision and adaptability. The bat algorithm and PSO can also serve as effective alternatives in specific contexts, where their specific characteristics can be leveraged: bat algorithm for initial exploration in spaces with many obstacles and PSO for continuous refinement. The structural limitations of the WOA restrict its applicability but it remains a viable option for simple or cost-sensitive disinfection tasks.

Ultimately, this work provides a practical guide for deploying autonomous UVC disinfection robots in various real-world environments, such as healthcare facilities, public transportation, industrial settings, large public spaces, or small offices, where optimized path planning ensures safety, efficiency, and health.

## 8. Future Work

While this study highlights the effectiveness of bio-inspired algorithms in optimizing UVC disinfection paths, certain limitations open opportunities for further research. A significant observation is that some algorithms, such as the WOA in the complex scenario, may occasionally converge to a local minima. This suggests that other algorithms might also encounter similar challenges when applied to different tasks. To address this, it is recommended to employ multiple algorithms either simultaneously or sequentially, closely monitoring their behavior throughout the optimization process and implementing mechanisms to recover from stagnation. These strategies are particularly critical in real-time applications, where dynamic adjustments or algorithm switching can play a key role in maintaining optimal performance. This approach also aligns with the concept of hybrid optimization frameworks, which capitalize on the strengths of multiple algorithms by dynamically adjusting parameters or transitioning between methods when stagnation is detected. Although such strategies are beyond the scope of this work, their potential makes them a compelling direction for future research in this field.

## Figures and Tables

**Table 1:** Low-complexity scenario computational time results.

Technique	Non-Coll. Time (NUP)	Non-Coll. Time (DP-BT)	Non-Coll. Time (DP-AT)	Opt. Sol Time
GOA	50 s	50 s	50 s	2 h 59 min 1 s
WOA	9 s	1 min 4 s	1 min 4 s	2 h 53 min 7 s
BA	13 s	13 s	13 s	1 h 41 min 52 s
PSO	34 s	2 min 49 s	4 min 17 s	2 h 58 min 51 s

**Table 2:** Low-complexity scenario iterations results.

Technique	Non-Coll. It (NUP)	Non-Coll. It (DP-BT)	Non-Coll. It (DP-AT)	Iterations
GOA	215	215	215	17,829
WOA	73	430	430	4144
BA	19	19	19	2707
PSO	216	665	863	3574

**Table 3:** Optimized trajectory results for the low-complexity scenario.

Technique	Opt. Path Time (s)	Opt. Path Distance (m)	Opt. Path Coverage (%)	Opt. Path Disinfection (%)	Max Radiation (J)
GOA	764.4583	36.2803	100%	100%	24,636
WOA	670.6846	20.6307	100%	100%	64,468
BA	444.5958	28.6626	100%	100%	35,366
PSO	185.1767	22.3233	100%	100%	20,750

**Table 4:** High-complexity scenario computational time results.

Technique	Non-Coll. Time (NUP)	Non-Coll. Time (DP-BT)	Non-Coll. Time (DP-AT)	Opt. Sol Time
GOA	34 min 18 s	34 min 18 s	1 h 44 min 27 s	2 h 2 min 7 s
WOA	1 min 36 s	-	-	-
BA	14 min 55 s	14 min 55 s	14 min 55 s	2 h 32 min 43 s
PSO	10 min 42 s	10 min 42 s	10 min 42 s	2 h 43 min 17 s

**Table 5:** High-complexity scenario computational iteration results.

Technique	Non-Coll. It (NUP)	Non-Coll. It (DP-BT)	Non-Coll. It (DP-AT)	Iterations
GOA	3523	3523	11,476	13,185
WOA	206	-	-	-
BA	1240	1240	1240	1753
PSO	728	728	728	1108

**Table 6:** Optimized trajectory results for the high-complexity scenario.

Technique	Opt. Path Time	Opt. Path Distance	Opt. Path Coverage (%)	Opt. Path Disinfection (%)	Max Radiation (J)
GOA	1.4171 × 10^3^	166.5824	100%	100%	14,518
WOA	-	-	2.7403%	0.40431%	447,930
BA	1.5748 × 10^3^	200.3029	100%	100%	163,300
PSO	1.5440 × 10^3^	191.8879	100%	100%	181,760

## Data Availability

Data are available upon reasonable request.

## Abbreviations

**Nomenclature**	**Description**
UVC	Ultraviolet type C
GOA	Gazelle Optimization Algorithm
WOA	Whale Optimization Algorithm
BA	Bat Algorithm
PSO	Particle Swarm Optimization
CCPP	Complete Coverage Path Planning
A*	A-Star Algorithm
RRT	Rapidly Exploring Random Tree
GA	Genetic Algorithm
PS	Pattern Search
GWO	gray Wolf Optimization
ACO	Ant Colony Optimization
ABC	Artificial Bee Colony
DNA	Deoxyribonucleic Acid
RNA	Ribonucleic Acid
UV	Ultraviolet
ROS	Robot Operating System
ETH-UDP	Ethernet User Datagram Protocol
ETH-REST	Ethernet Representational State Transfer
LIDAR	Light Detection and Ranging
DI/DO	Digital Input/Digital Output
MAE	Mean Absolute Error
AGV	Automated Guided Vehicle
CC	Collision Condition
NCC-NDP	Non-Collision Condition With Non-Distributed Path
NCC-DPBT	Non-Collision Condition With Distributed Path Below Threshold
NCC-DPAT	Non-Collision Condition With Distributed Path Above Threshold
NUP	Non-Uniform Path
DB-BT	Distributed Path—Below Threshold Radiation
DP-AT	Distributed Path—Above Threshold Radiation
